# Cognitive Fatigue Destabilizes Economic Decision Making Preferences and Strategies

**DOI:** 10.1371/journal.pone.0132022

**Published:** 2015-07-31

**Authors:** O’Dhaniel A. Mullette-Gillman, Ruth L. F. Leong, Yoanna A. Kurnianingsih

**Affiliations:** 1 Department of Psychology, National University of Singapore, Singapore, Singapore; 2 Centre for Cognitive Neuroscience, Neuroscience and Behavioral Disorders Program, Duke-NUS Graduate Medical School, Singapore, Singapore; 3 SINAPSE Institute for Cognitive Science and Neurotechnologies, Singapore, Singapore; Brain and Spine Institute (ICM), FRANCE

## Abstract

**Objective:**

It is common for individuals to engage in taxing cognitive activity for prolonged periods of time, resulting in cognitive fatigue that has the potential to produce significant effects in behaviour and decision making. We sought to examine whether cognitive fatigue modulates economic decision making.

**Methods:**

We employed a between-subject manipulation design, inducing fatigue through 60 to 90 minutes of taxing cognitive engagement against a control group that watched relaxing videos for a matched period of time. Both before and after the manipulation, participants engaged in two economic decision making tasks (one for gains and one for losses). The analyses focused on two areas of economic decision making—preferences and choice strategies. Uncertainty preferences (risk and ambiguity) were quantified as premium values, defined as the degree and direction in which participants alter the valuation of the gamble in comparison to the certain option. The strategies that each participant engaged in were quantified through a choice strategy metric, which contrasts the degree to which choice behaviour relies upon available satisficing or maximizing information. We separately examined these metrics for alterations within both the gains and losses domains, through the two choice tasks.

**Results:**

The fatigue manipulation resulted in significantly greater levels of reported subjective fatigue, with correspondingly higher levels of reported effort during the cognitively taxing activity. Cognitive fatigue did not alter uncertainty preferences (risk or ambiguity) or informational strategies, in either the gains or losses domains. Rather, cognitive fatigue resulted in greater test-retest variability across most of our economic measures. These results indicate that cognitive fatigue destabilizes economic decision making, resulting in inconsistent preferences and informational strategies that may significantly reduce decision quality.

## Introduction

Cognitive fatigue is a ubiquitous human condition, the result of sustained cognitive engagement that taxes our mental resources. The nature of work in our society is changing such that increasingly, work involves demanding cognitive activity, as opposed to physical exertion, with working hours no longer restricted by daylight. Demanding work schedules lead many people to experience cognitive fatigue on a daily basis, and have resulted in high burnout rates [1,2]. Studies examining effects of such fatigue find that persistent mental resource burdens result in diminished motivation, increased distractibility, changes in information processing and poorer mood [3–10]. Moreover, fatigued participants are more likely to fail to detect errors and less likely to take remedial action, and are more willing to take chances in everyday decision making [11]. Such general deficits can easily lead to diminished performance and health, such as progressive impairment of treatment decisions by doctors [12].

We sought to specify the impact of cognitive fatigue on economic decision making, with a focus on uncertainty preferences and strategy. Uncertainty refers to the absence of information about the eventual resolution of probabilistic events, such as in gambles. The two common forms of uncertainty are risk, which involves known probabilities (e.g., a coin flip), and ambiguity, which pertains to probabilities of outcomes that are unknown or cannot be estimated [13–15].

Beyond economic preferences, we also examined what information the participant utilized to inform their choices [16]. We contrasted between two dominant types of information presented in each trial, corresponding to maximizing and satisficing strategies. Maximizing features calculation of the relative expected value of the two options, the determination of which requires multiple mathematical calculations. Satisficing focuses on the probability of winning, which is simply visually observed as the proportion of a circle segment. These strategies differ in terms of cognitive cost, with satisficing less cognitively taxing than maximizing.

A priori, we hypothesized that cognitive fatigue would result in increased satisficing strategies, as fatigued participants opt for less effortful strategies over those requiring more mental resources. This was based on two directions of prior findings. First, studies suggest that cognitive fatigue results in compromised top-down control mechanisms with relative sparing of automatic processes [17]. Secondly, aversion to further effort is a common feature of mental fatigue [10,18–20]. Fatigued individuals may seek to minimize the energetic costs by opting for strategies that require lower levels of effort [21].

We investigated the impact of cognitive fatigue on economic decision making, specifically alterations of uncertainty preferences and/or choice strategies. We employed two incentive-compatible economic decision making tasks, examining for alterations in economic decision making independently across both the gains and the losses domains. We utilized a between-subjects design, comparing participants that engaged in a cognitively taxing task (fatigue group) and those that watched relaxing videos for an equivalent period of time (control group). We found that cognitive fatigue did not produce a reliable shift in individual uncertainty preferences or choice strategies, contrary to our initial hypotheses. Rather, cognitive fatigue resulted in significantly greater test-retest variability across multiple measures. These results indicate that cognitive fatigue results in the destabilization of economic decision making, highlighting the dangers of fatigue.

## Materials and Methods

### Participants

All participants provided written informed consent under a protocol approved by the National University of Singapore Institutional Review Board (IRB). Our sample consisted of 76 university students (41 males) with an age range of 19–26 years (*M* = 22.3 years, *SD* = 1.74), recruited through advertisements at the National University of Singapore. Data were collected in 2 samples. In the initial sample, 44 participants took part in the study and were randomly assigned to a fatigue (*N* = 19) or control (*N* = 25) conditions. Examining the fatigue manipulation for these participants indicated that although the fatigue condition reported cognitive fatigue and significantly higher levels of cognitive effort expended, there was no decline in performance in the manipulation task. While this effect has been previously reported, we opted to enhance the cognitive fatigue manipulation to ensure that our manipulation was sufficient [22]. The second sample of data collection was an additional 32 participants in an extended fatigue condition, with the intention of merging the two samples of fatigue groups if they were not statistically different.

Four participants were excluded from analyses within the fatigue group, as they did not report successful inducement of cognitive fatigue following the manipulation task. Our final sample was 25 participants (11 males; *M*: 21.9 years, *SD* = 1.44) in the control condition and 47 participants (28 males; *M*: 22.5 years, *SD* = 1.87) in the fatigue condition. All participants were asked to abstain from alcohol and caffeine 24 hours before the experiment.

### Study procedure

**Fig 1:**
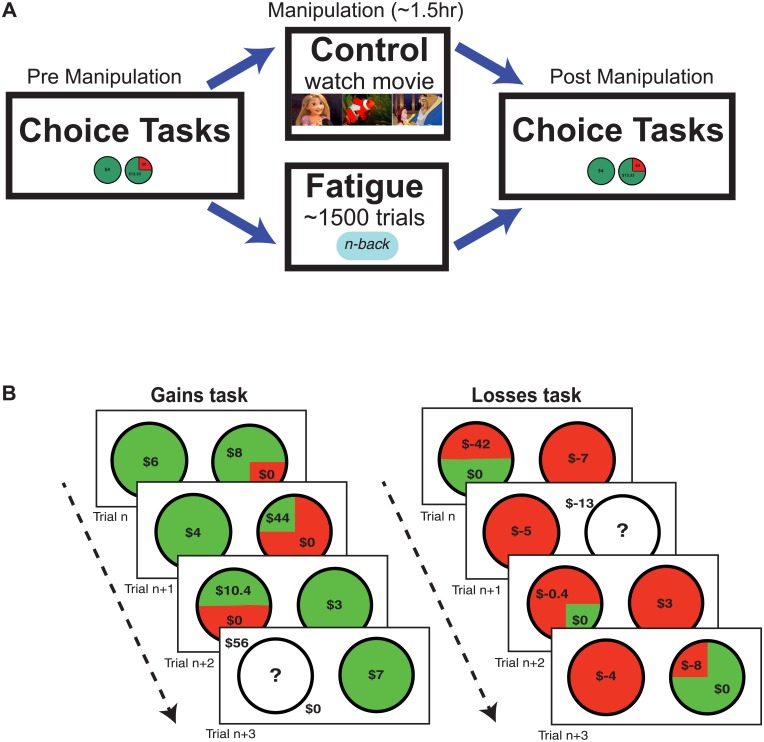
Experimental procedures and tasks. **(A)** All participants filled in the initial questionnaires, followed by a pre-manipulation risk task. In the manipulation period, participants in the fatigue condition performed 5–7 blocks of 300 trials of the N-back task to induce fatigue. Participants in the control condition spent an equivalent amount of time (approx. 90min) watching relaxing videos. After the manipulation phase, all participants performed the risk task again. **(B)** The economic decision making task comprised of gains and losses domains, whereby participants were required to choose between a certain or gamble option. They were given no time limit to respond. Participants were paid based on random selection and resolution of one trial from each domain after the completion of the entire experiment.

Each experimental session lasted approximately 2.5 hours, for which participants were paid $10 plus an additional $0 to 10 (dependent on the resolution of one randomly selected trial for each choice task). Following IRB consent, each session proceeded in the following order: 1) initial questionnaires, 2) pre-manipulation phase, 3) manipulation phase, 4) post-manipulation phase (see Fig 1A). At the beginning of the session, participants were briefed on the procedure and asked to perform at their best.

#### Initial questionnaires

Participants filled in questionnaires on their demographics, their recent health status, a self-reported cognitive fatigue question, and the Rating Scale Mental Effort (RSME) to assess cognitive fatigue (subjective fatigue measures; see section below).

#### Pre-manipulation phase

Next, all participants completed the two computerized economic decision making tasks. Participants were informed that one trial of each task would be randomly selected and resolved at the end of the whole experiment to determine their total payment. Importantly, no gambles were resolved until the completion of the experiment to prevent behavioral alterations due to outcome feedback (i.e., learning effects).

#### Manipulation phase

The manipulation phase followed, with differential treatments for the participants in the fatigue and control conditions. Participants in the fatigue condition performed a cognitively demanding N-back task (5–7 blocks of 300 trials, 2-back) for approximately 90 minutes, while participants in the control group spent 90 minutes watching relaxing videos of nature and animated cartoons. Participants in the non-fatigue group were told to relax and enjoy the videos. The aim was to keep them sufficiently engaged such that they would not be drowsy, but rather, in a neutral state of wakefulness. They were provided with a menu of 3 videos that they could freely switch between: 1) BBC’s ‘The Life of Birds’, 2) Disney’s ‘Beauty and the Beast’, 3) Disney’s ‘Tangled’. All participants were limited to water (no food or other drinks) for the duration of the study.

#### Post-manipulation phase

Participants completed the self-reported cognitive fatigue question and RSME scale. Participants then repeated the two computerized economic decision making tasks. Participants were reminded that one trial from each task would be randomly selected and resolved at the end of the experiment to determine their final compensation.

At the end of the session, one trial from each of the 4 economic tasks were randomly selected and resolved. Participants were presented with their earnings and provided the opportunity to ask any questions they might have about the experiment.

### Experimental design

#### Subjective fatigue measures

**Fig 2:**
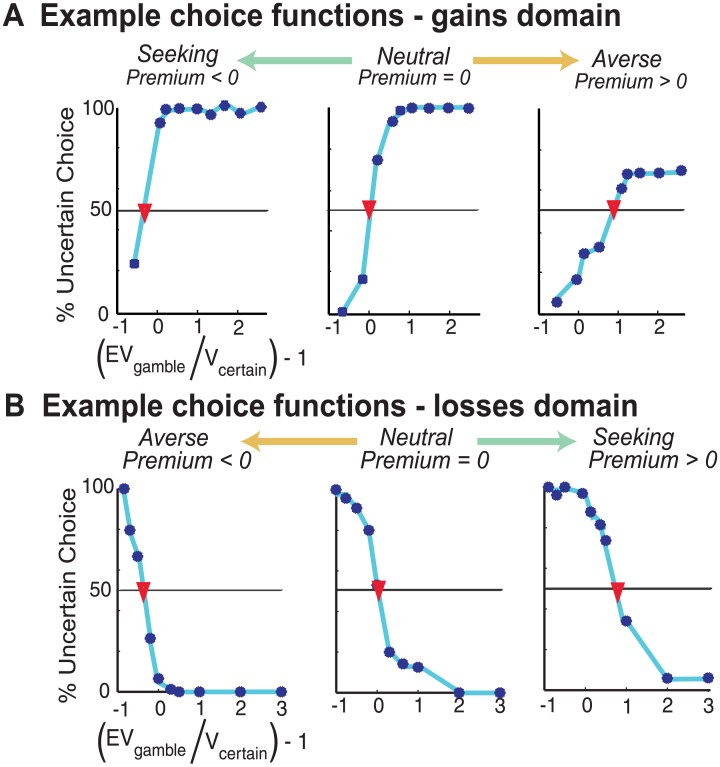
Cognitive fatigue ratings. State fatigue and effort across the experimental protocol. The fatigue (orange bars) and control groups (light and dark blue bars: 5-blocks and 7-blocks of N-back respectively) did not differ significantly in **(A)** self-reported cognitive fatigue pre- manipulation and **(B)** RSME scores, at baseline. However, post- manipulation, the fatigue group reported significantly higher cognitive fatigue and RSME scores as compared to the non-fatigue group, suggesting that the manipulation was successful in inducing fatigue in the fatigue groups.

Throughout the experiment, participants filled in two short questionnaires probing state subjective fatigue and effort (below) (see Fig 2). The initial questionnaires were filled in with the initial demographic questionnaires, the second after the pre-manipulation economic task, the third to seventh (or ninth) were filled in after each block of the N-back task, and the last questionnaire was filled in after the post-manipulation economic tasks.

#### RSME

The Rating Scale of Mental Effort (RSME) measures the subjective amount of effort participants have engaged in [23]. It is a state measure consisting of one question, asking the respondent how much effort he or she had to invest to perform the activities just completed. This scale is scored from 0 to 150, with verbal anchors of ‘absolutely no effort’ to ‘extreme effort’, respectively. The RSME has been used in numerous studies investigating cognitive fatigue, with significant increases in reported cognitive effort invested interpreted as indicating cognitive fatigue [17].

#### Self-reported fatigue

Direct self-reported fatigue was assessed with the question of “How cognitively fatigued are you?” with responses on a 10-point scale ranging from 1-not at all to 10-extremely fatigued.

#### Cognitive fatigue manipulation: N-back task

Cognitive fatigue was induced in participants through completion of 1500 or 2100 trials of the N-back task (5–7 blocks of 300 trials), over approximately 60 to 90 minutes (depending on number of blocks). The N-back task is often associated with studies of working memory, and has been shown to tax working memory [22,24–26]. On each trial, participants were required to respond with a right button press for the target stimulus and a left button press for any non-target stimulus. We utilized a 2-back condition, in which targets were defined as the letter that is the same as the letter that was presented two trials prior to the current trial. Stimuli were letters of the alphabet, and the full range of letters was used. Each trial was 2 seconds (consisting of 500 ms presentation of white letters in the middle of a black screen and 1.5 seconds for response and feedback), with an inter-trial interval of 200 ms. These values resulted in approximately 15 minutes for each block, with approximately 12 minutes of active task (plus additional time to fill out the pen-and-paper effort and fatigue questionnaires, and restart the task). The task was performed on a computer using MATLAB (v7.10.0, Mathworks, Inc.) and Psychtoolbox extensions (v3)[27–29].

#### Economic decision making tasks

We employed two computerized economic decision making tasks, the first relating to the domain of gains (gains choice task), and the second relating to the domain of losses (losses choice task)[16,30]. On each trial, participants chose between a certain option and one of the uncertain options (risky or ambiguous).

The gains choice task consisted of 135 risky trials and 30 ambiguous trials intermixed with each other. The amount of money offered by the certain option for both risky and ambiguous trials ranged from $3 to $7. In the risky trials, participants were offered a gamble of known probability with three possible probabilities of winning (25%, 50% and 75%) and nine relative expected values (ratio of expected value of the gamble over the value of the certain option; 0.5, 1.0, 1.3, 1.6, 1.9, 2.2, 2.5, 3.0 and 3.5). These resulted in potential winnings from $2 to $98. In the ambiguous trials, participants were offered a gamble with an undetermined probability, having been told that we would randomly select a probability (from 0 to 1) before randomly resolving the gamble. For ambiguity trials, we examined six ratios of expected value (0.5, 1.0, 2.0, 3.0, 4.0 and 6.0), calculated using a 50% probability based on the law of large numbers. These resulted in potential winnings from $2 to $168.

The losses choice task mirrored the gains choice task save for alteration of the sign of the value of the options (certain options are certain losses, and gambles feature a potential loss against a potential zero outcome), and adjustment of the EV_G_/V_C_ to provide a greater density of rEV values below 1. Ten relative expected values were examined for both risky and ambiguous trials (0.1, 0.3, 0.5, 0.8, 1.0, 1.3, 1.5, 2.0, 3.0 and 4.0). These resulted in 150 risky trials and 50 ambiguous trials, with potential losses ranging from $0.40 to $112 for risk trials and ambiguous trials. Fig 1B illustrates trials encountered by participants in both the gains and losses domains.

Prior to beginning either task, participants were reminded that their final monetary compensation would include a proportion of the total monies they gather from resolving a randomly selected trial from each task (four altogether; two tasks in the pre-manipulation phase and two tasks in the post-manipulation phase). Both tasks were self-paced for all trials.

### Uncertainty preference metrics

Across both the gains choice task and the losses choice task, we were interested in examining the effects of cognitive fatigue on four different uncertainty preferences—risk in gains, ambiguity in gains, risk in losses, and ambiguity in losses. With the performance of each task both before and after the manipulation, this resulted in a total of 8 preference values for each participant.

**Fig 3:**
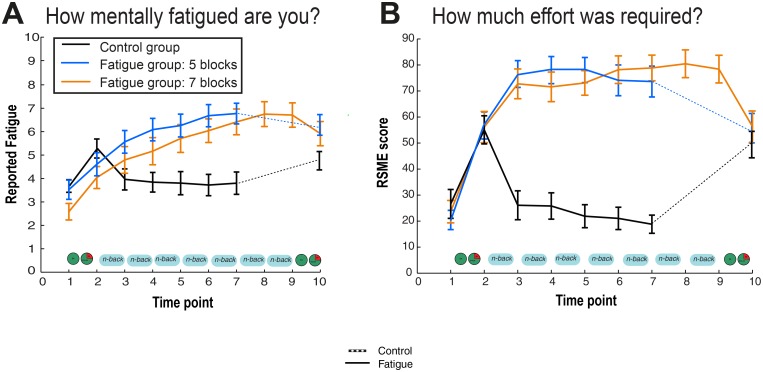
Example choice functions. **(A)** In the gains domain, the range of risk preferences is represented on a continuum from risk seeing (left) to risk averse (right). The indifference point of each choice function is marked with a red inverted-triangle. Risk premium is determined by the value on the ‘(rEV_G_ / V_c_) -1’ (x-axis) at this indifferent point. **(B)** In the losses domain, the range of risk preferences is represented on a continuum from risk averse (left) to risk seeking (right).

Each preference value was determined through psychometric determination of the degree to which participants altered the value of that type of gamble, relative to the certain option [30]. To derive this metric, choice functions were constructed by plotting the percentage of choices of the uncertain option as a function of the ratio of the expected value of the gamble to the value of the certain option (EV_G_/V_G_) (see Fig 3 for example choice functions). An indifference point was determined as the first point at which the choice function crossed 50%, indicating the ratio at which the participant was indifferent between the gamble and certain options. This indifference point was modified by subtracting 1 to produce a premium metric—a measure of how participants alter the expected value of a gamble option due to the probabilistic outcome being unknown. Within both domains, a premium value of 0 indicates neutrality. Within the gains domain, a positive premium indicates aversion (devaluing the gamble) and a negative premium value indicates seeking (increasing the value of the gamble). The relationships are inverted in the losses domain, resulting in positive premium values indicating seeking and a negative premium value indicating aversion.

Of note, a small number of participants presented choice functions that did not cross the 50% mark. We are unable to calculate the preference values for such choice functions. Furthermore, we cannot differentiate between whether these participants were engaging their preferences or simply relying on satisficing heuristics (such as always choosing the certain option) (for further discussion, see Stanton et al., 2011 and Kurnianingsih et al., 2015) [16,30]. As such, these participants were excluded from specific analyses, resulting in differential subject counts across tests. Final counts for each metric for each domain are shown in Table 1.

**Table 1:** Mean (standard deviation) differences in uncertainty premiums and information strategy metrics in the gains and losses domains between controls and fatigued subjects.

	Control		Fatigue		*p*
	Means (*SD*)	N	Means (*SD*)	N	
**Gains**					
Uncertainty Premium					
Risk	-.121 (.213)	14	-.248 (.721)	30	.524
Ambiguity	-.213 (.739)	13	-.417 (1.283)	27	.599
Information strategy					
Choice strategy	-.096 (.186)	23	-.096 (.205)	43	.995
rEV r^2^	-.031 (.108)	23	-.064 (.087)	46	.186
pWIN r^2^	.065 (.122)	23	-.028 (.143)	43	.299
**Losses**					
Uncertainty Premium					
Risk	.035 (.400)	23	-.090 (.582)	43	.364
Ambiguity	-.016 (.299)	23	-.006 (.545)	41	.937
Information strategy					
Choice strategy	-.005 (.135)	25	-.026 (.150)	45	.576
rEV r^2^	-.002 (.104)	25	-.015 (.122)	45	.647
pWIN r^2^	.003 (.049)	25	.010 (.047)	45	.555

Mean differences are calculated as post—pre.

In order to facilitate generalization and comparison of these results, we additionally calculated our participants’ pre-manipulation uncertainty preference using a power function metric, and tested the correlation of this metric with our premium metric. Previously, we have found very high correlations in these metrics across two samples of younger adults (N~300, r > |.7|; N = 62, r > |.7|) [16,30]. In the current sample, we found high correlations within both domains for both risk (Gains: *r*(48) = -0.497, *p* < 0.001; Losses: *r*(66) = -0.640, *p* < 0.0001) and ambiguity (Gains: *r*(38) = -0.773, *p* < 0.0001; Losses: *r*(62) = -0.877, *p* < 0.0001) preferences. These high correlations indicate clearly that these varying specific formulations of risk preferences are able to largely capture the same variance across participants.

### Choice strategy metric

We investigated the informational strategies that participants employed in making their decisions during the gains choice and losses choice tasks. Specifically, we were interested in contrasting the reliance on two dominant competing strategies, with one corresponding to maximizing behaviour and the other to satisficing. The maximizing strategy is to compute the ratio of the expected value of the gamble to the value of the certain option (rEV). The satisficing strategy is to simply rely upon the probability of winning the gamble (pWIN), which is evident in the proportion of the pie-shaped representation. Employing the maximizing strategy (rEV information) is more cognitively taxing as it requires mathematical calculations, but it is the optimal strategy, as on average it will result in higher outcomes. The use of the satisficing strategy (pWIN information) is much less effortful as it recruits readily available perceptual information (probability is inferred from coloured segments of the pie), but results in lower expected outcomes on average.

To compare the relative influence of these strategies for each participant, we determined how much of the variance in their choices (across trials) could be accounted for by each factor. This was done through independent linear regressions, which determines how much influence the variation in the examined factor across trials has on the choices made on those trials (in the r-squared value). Importantly, in this task, the value of the pWIN and rEV for each trial are orthogonal across trials (correlation is zero), so the independent regressions cannot result in these factors accounting for the same choice variance.

To compare how much each participant relied upon each of these competing strategies, we contrasted them directly by taking the difference in their r-squared values (rEV minus pWIN) to produce our ‘choice strategy’ metric. If choice strategy is positive, this indicates that a participant is maximizing (relying more on rEV information than pWIN information). If choice strategy is negative, the participant is satisficing (greater use of pWIN information over rEV information). A value of 0 would indicate that a participant is using the two types of information equally.

### Statistical analyses

All statistical analyses were performed with SPSS version 20 (IBM, US) and MATLAB (v7.10.0, Mathworks, Inc.). All statistical tests were two-tailed and the significance level was set at *p* < 0.05.

To compare the effects of our manipulation on the fatigue and control groups, we calculated a difference metric for each of our preference and choice strategy measures, taking the post minus pre scores.

## Results

### Fatigue samples were not statistically different

We collected two fatigue groups of participants for this experiment, varying the degree of the fatigue manipulation. The initial sample (*N* = 19) performed 5 blocks of 300 trials of the N-back task (~60 minutes), while the later sample (*N* = 32) performed 7 blocks of 300 trials of the N-back task (~90 minutes).

Comparing the initial and later sample, we found no significant differences in pre- or post- RSME manipulation scores or self-reported cognitive fatigue between the 5 blocks or 7 blocks manipulation groups (*p*_*RSME pre*_ = 0.705; *p*_*RSME post*_ = 0.391; *p*_*fatigue pre*_ = 0.080; *p*_*fatigue post*_ = 0.667). As participants did not self-report differences in fatigue or effort, we merged the two groups into a single fatigue group (*N* = 47) for all subsequent analyses.

### Performance on N-back task

Participants in the fatigue condition completed 5 or 7 blocks of 300 trials of the N-back task (Table 2). To examine whether performance declined over blocks, we compared block 4 (peak performance) and the final block (block 5 or 7) on measures of percentage of correct trials, misses and false alarms. Paired t-tests revealed no significant difference for misses and false alarms between block 4 and the final block (misses: *t*(48) = -.382, *p* = 0.704); false alarms: *t*(47) = -1.263, *p* = 0.213), and a trend toward significance for percentage of correct trials (*t*(48) = 1.840, *p* = .072). Overall, performance was maintained across trials.

**Table 2:** Mean (standard deviation) performance on N-back task from block 1 to 7.

	Block 1	Block 2	Block 3	Block 4	Block 5	Block 6	Block 7
N	51	51	51	51	49	25	25
Correct (%)	73.12 (16.07)	83.73 (14.34)	85.07 (13.20)	85.46 (13.84)	84.79 (14.63)	85.65 (11.88)	86.81 (12.65)
Misses (%)	10.31 (5.46)	7.14 (5.69)	6.58 (5.08)	7.28 (6.35)	7.02 (5.83)	8.23 (7.11)	7.37 (7.37)
False alarms (%)	7.66 (5.07)	5.39 (3.81)	5.58 (4.25)	4.97 (5.25)	5.78 (5.87)	5.07 (4.76)	5.29 (5.02)
Response	.74	.68	.63	.61	.60	.58	.58
time (s)	(.30)	(.31)	(.25)	(.24)	(.25)	(.14)	(.13)

Response times are the mean of individual medians, in seconds.

### Cognitive fatigue manipulation check: higher effort and fatigue

Pre-manipulation, the fatigue and control groups presented similar baseline levels, with no significant differences in their RSME scores or self-reported cognitive fatigue levels (RSME, fatigue group, *M* = 22.3, *SD* = 18.5; control group, *M* = 26.6, *SD* = 27.2; *t*(69) = 0.783, *p* = 0.436) (Fig 2B) (self-reported fatigue, fatigue group: *M* = 3.00, *SD* = 1.85; control group: *M* = 3.68, *SD* = 1.31; *t*(69) = 1.626, *p* = 0.108) (Fig 2A).

To ensure the cognitive fatigue manipulation was overall effective, we examined the change in RSME scores and self-reported cognitive fatigue levels across groups. Change values were calculated as the differences between the last values prior to the second economic task minus their initial reported values. Significant differences were found across our two subject groups, with significantly higher levels of effort and resultant cognitive fatigue in the manipulation group (RSME, *t*(65) = 8.78, *p* < 0.0001; cognitive fatigue, *t*(69) = 7.57, *p* < 0.0001).

To examine whether there were differences between fatigue and control groups RSME scores we ran two repeated measures ANOVAs with Greenhouse-Geisser correction comparing across the 6 time points where effort and fatigue levels were measured (initial and each subsequent ~15 minutes, between blocks for the fatigue group). For the first, examining RSME scores (self-reported effort), we found significant differences across time points between the fatigue and control groups (*F*(2.85, 182.32) = 44.38, *p* < 0.0001). Post-hoc t-tests with full-Bonferroni correction to account for the 6 between-group comparisons (adjusted threshold *p* = .008) revealed no difference at the initial time point between fatigue and control groups (pre-manipulation, *t*(67) = -0.162, *p* = 0.872), but significant differences for all subsequent time points (*t* > 7.50, *p* < 0.0001).

For the second, examining self-reported cognitive fatigue levels, we also found significant differences across time points for fatigue levels between the fatigue and control groups (*F*(2.36, 162.78) = 37.89, *p* < 0.0001). The pattern was very similar to that found prior, with no significant difference between groups at the initial or second time point (full-Bonferroni adjusted threshold *p* = 0.008; initial, *t*(69) = 1.95, second, *p* = 0.056; *t*(69) = 1.90, *p* = 0.062), and significant differences between groups for all subsequent time points (*t* > 2.89, *p* < 0.006).

Overall, these results demonstrate that prolonged performance on the N-back task successfully induced a state of cognitive fatigue, in accordance with previous reports [22,31].

### Relationship between subjective and objective fatigue

Within the fatigue group, we examined whether there were relationships between the change in self-reported levels of fatigue (and effort) and performance on the N-back task (correct %, miss %, false alarm %, and response time)—examining if there are relationships between objective and subjective fatigue. For each of these measures, we calculated the change measure by subtracting the value in block 1 from the value in block 5. We used a full-Bonferroni correction to account for the 4 correlations performed on each of the subjective measures of fatigue (change in fatigue and change in effort), resulting in a threshold of *p* = 0.0125. None of the relationships were found to be significant.

### Cognitive fatigue does not shift economic decision making

To examine the effects of cognitive fatigue on our economic decision making metrics (preferences and choice strategies), we compared the mean difference in changes in economic measures (post-pre) between the fatigue and control group (Tables 1 and 3).

**Table 3:** Pre- and post- manipulation means (standard deviations) in uncertainty premiums and information strategy metrics in the gains and losses domains for both controls and fatigued subjects.

	Control				Fatigue			
	Pre	N	Post	N	Pre	N	Post	N
**Gains**								
Uncertainty Premium								
Risk	.554 (.607)	15	.518 (.587)	16	.810 (.853)	35	.422 (.797)	31
Ambiguity	1.392 (1.884)	15	1.007 (1.003)	14	1.530 (1.250)	28	1.013 (1.244)	29
Information strategy								
Choice strategy	.042 (.330)	24	-.048 (.391)	24	.064 (.270)	46	-.0495 (.243)	44
rEV r^2^	.221 (.169)	24	-.194 (.176)	24	.250 (.145)	46	-.050 (.243)	44
pWIN r^2^	.180 (.184)	24	.241 (.234)	24	.142 (.155)	46	.175 (.164)	44
Response time (s)	1.665 (.623)	25	1.187 (.418)	25	1.564 (.606)	47	.1075 (.399)	47
**Losses**								
Uncertainty Premium								
Risk	.192 (.419)	23	.236 (.579)	24	.120 (.498)	45	.039 (.487)	43
Ambiguity	.040 (.337)	23	.024 (.353)	23	.010 (.385)	42	-.004 (.573)	42
Information strategy								
Choice strategy	.387 (.210)	25	.382 (.187)	25	.359 (.128)	46	.330 (.147)	45
rEV r^2^	.435 (.139)	25	.433 (.129)	25	.380 (.120)	47	.369 (.122)	45
pWIN r^2^	.047 (.082)	25	1.356 (.367)	25	.1723 (.569)	47	1.176 (.396)	46
Response time (s)	1.878 (.600)	25	1.356 (.368)	25	1.723 (.569)	47	1.176 (.401)	46

Response times are the mean of individual medians.

#### Cognitive fatigue does not shift response times

Cognitive fatigue did not alter response times in either the gains or losses domains. We found no significant main effect of fatigue on response time within either the gains choice task (t-test, Control: *M*_*diff*_ = -0.48, *SD*_*diff*_ = 0.48; Fatigue: *M*_*diff*_ = -0.49, *SD*_*diff*_ = 0.41; *t*(70) = 0.110, *p* = 0.913) or the losses choice task (Control: *M*_*diff*_ = -0.52, *SD*_*diff*_ = 0.40; Fatigue: *M*_*diff*_ = -0.53, *SD*_*diff*_ = 0.40; *t*(69) = 0.112, p = 0.911) (Tables 1 and 3).

#### Cognitive fatigue does not shift risk or ambiguity preferences

For each metric, we calculated the change in preference or strategy (post-pre) and compared between the fatigue and control groups with a simple independent sample t-test (Table 1). We excluded one participant as an outlier in the gains domain analyses, as their risk difference value (risk premium_post_−risk premium_pre_) was > 5*SD* from the mean (*M* = 0.198, *SD* = 0.363) (Tables 1 and 3).

**Fig 4:**
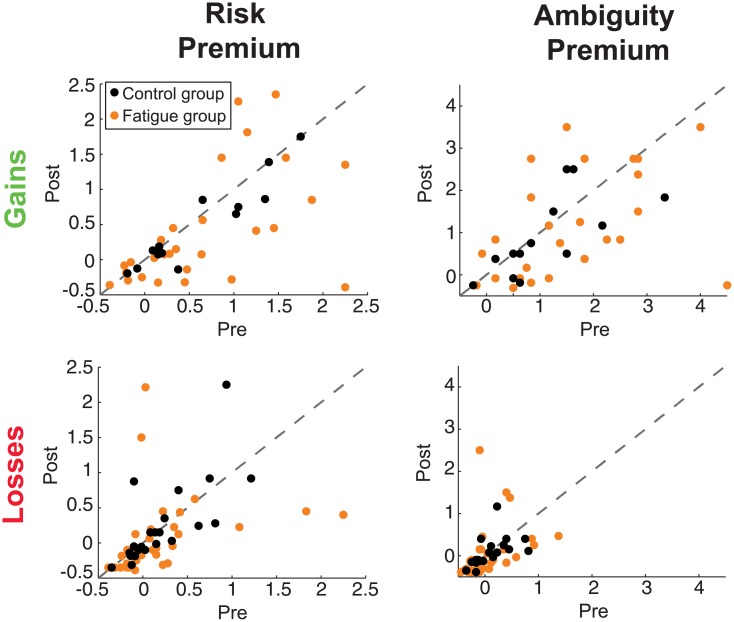
Uncertainty preferences in the gains and losses domains. Relationship between pre- and post- manipulation risk and ambiguity premiums values for fatigue (blue) and control (orange) groups in the gains and losses domains.

Within the gains domain, we found no significant main effect of fatigue on risk premium (Control: *M*_*diff*_ = -0.12, *SD*_*diff*_ = 0.21; Fatigue: *M*_*diff*_ = -0.12, *SD*_*diff*_ = 0.21; *t*(42) = 0.642, *p* = 0.542) or ambiguity premium (Control: *M*_*diff*_ = -0.21, *SD*_*diff*_ = 0.74; Fatigue: *M*_*diff*_ = -0.42, *SD*_*diff*_ = 1.28; *t*(38) = 0.531, *p* = 0.599) (Table 1 and Fig 4).

Within the losses domain, similarly, we found no significant main effect of fatigue on risk premium (Control: *M*_*diff*_ = 0.04, *SD*_*diff*_ = 0.40; Fatigue: *M*_*diff*_ = -0.90, *SD*_*diff*_ = 0.58; *t*(64) = 0.915, *p* = 0.364) or ambiguity premium (Control: *M*_*diff*_ = -0.02, *SD*_*diff*_ = 0.30; Fatigue: *M*_*diff*_ = -0.01, *SD*_*diff*_ = 0.55; *t*(62) = 0.080, *p* = 0.937) (Table 1 and Fig 4).

#### Cognitive fatigue does not shift choice strategy

Analyses of choice strategy metrics revealed a similar pattern as with the preference metrics. Within the gains domain, there were no significant differences in the change in choice strategy between the fatigue and control groups (Control: *M*_*diff*_ = -0.10, *SD*_*diff*_ = .19; Fatigue: M_diff_ = -0.10, *SD*_*diff*_ = 0.21; *t*(64) = 0.006, *p* = 0.995) (Table 1 and Fig 4).

As the choice strategy metric is a composite, and a lack of difference in the composite measure does not mean there is no change in the components, we also examined whether there were any significant differences in the use of rEV and pWIN information separately. Concurring with the composite measures, we found no significant effect of cognitive fatigue on either component (rEV r^2^ [Control: *M*_*diff*_ = -0.03, *SD*_*diff*_ = 0.11; Fatigue: *M*_*diff*_ = -0.06, *SD*_*diff*_ = 0.09; *t*(67) = 1.338, *p* = 0.186] and pWIN r^2^ [Control: *M*_*diff*_ = 0.07, *SD*_*diff*_ = 0.12; Fatigue: *M*_*diff*_ = 9.03, *SD*_*diff*_ = 0.14; *t*(64) = 1.048, *p* = 0.299]).

The same pattern of results were found in the losses domain (Table 1 and Fig 4), with no significant difference in the change in choice strategy between the two groups (Control: *M*_*diff*_ = -0.01, *SD*_*diff*_ = 0.14; Fatigue: *M*_*diff*_ = -0.03, *SD*_*diff*_ = 0.15; *t*(68) = 0.562, *p* = 0.576) and no alterations of the independent components (rEV r^2^ [Control: *M*_*diff*_ = -0.002, *SD*_*diff*_ = 0.104; Fatigue: *M*_*diff*_ = -0.02, *SD*_*diff*_ = 0.12; *t*(68) = 0.460, *p* = 0.647] and pWIN r^2^ [Control: *M*_*diff*_ = 0.003, *SD*_*diff*_ = 0.05; Fatigue: *M*_*diff*_ = 0.01, *SD*_*diff*_ = 0.05; *t*(68) = 0.594, *p* = 0.555]).

### Cognitive fatigue reduces the stability of economic decision making

**Fig 5:**
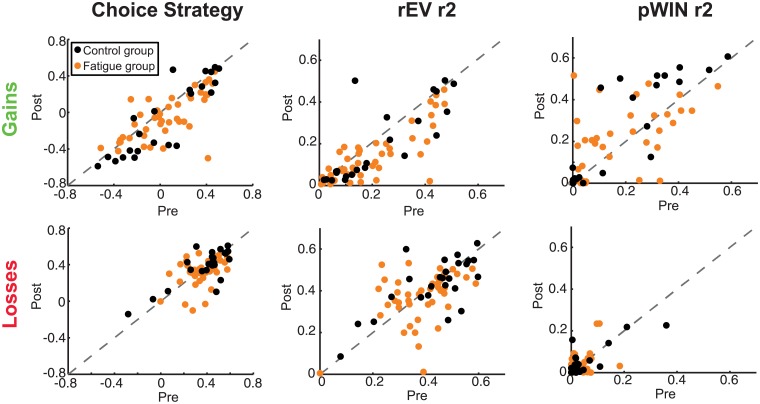
Choice strategies in the gains and losses domains. Relationship between pre- and post- manipulation independent r-squared values of **(A)** strategy, **(B)** rEV and **(C)** pWIN on trial-by-trial choice behaviour for both fatigue and control groups.

Post-hoc, we noticed a pattern of reduced test-retest correlations within the fatigue group across the economic decision making measures. To quantify this, we used a Fisher’s r-to-z transformation to compare the pre- and post- correlations between fatigue and control groups for each metric (Table 4 and Fig 5).

**Table 4:** Test-retest correlations for uncertainty premiums and information strategy metrics in the gains and losses domains for controls and fatigued subjects.

	Control		Fatigue			
	*r*	*p*	*r*	*p*	*z*	*p*
**Gains**						
Uncertainty Premium						
Risk	.94	< .0001	.56	< .001	3.10	**.002**
Ambiguity	.69	< .01	.48	.011	.860	.389
Information strategy						
Choice strategy	.89	< .0001	.68	< .0001	2.07	**.039**
rEV r^2^	.81	< .0001	.80	< .0001	0.11	.912
pWIN r^2^	.70	< .0001	.60	< .0001	0.41	.682
**Losses**						
Uncertainty Premium						
Risk	.74	< .0001	.32	.039	2.24	**.025**
Ambiguity	.63	< .01	.41	< .01	1.13	.259
Information strategy						
Choice strategy	.77	< .0001	.41	< .001	2.29	**.022**
rEV r^2^	.70	< .0001	.45	< .01	1.45	.147
pWIN r^2^	.80	< .0001	.44	< .01	2.42	**.015**

*r*: correlation coefficient; *z*: Fisher’s r-to-z transformation test for the significance of the difference between two correlation coefficients.

#### Cognitive fatigue reduces the stability of risk preferences

Risk premium values in the gains domain showed significantly lower test-retest correlations in the fatigue group than the control group (*z* = 3.10, *p* = 0.002). This effect was not found for the ambiguity premiums (*z* = 0.86, *p* = 0.389). The losses domain mirrored the results of the gains domain; risk premium test-retest correlations were significantly lower in the fatigue group than in the control group (*z* = 2.24, *p* = 0.025), with no difference for ambiguity premiums (*z* = 1.13, *p* = 0.259).

#### Cognitive fatigue reduces the stability of choice strategy

Choice strategy values, in both the gains and the losses domains, had significantly lower test-retest stability in the fatigue group than in the control group (gains, *z* = 2.07, *p* = 0.039; losses, *z* = 2.29, *p* = 0.022) (Table 4 and Fig 5).

### Change in fatigue does not relate to individual change in economic metrics

It is possible that individual changes in fatigue or effort could account for individual differences in the economic metrics. For example, while there is no mean shift in risk preference across groups, it is still possible that there could be a linear relationship between individual change in fatigue and individual change in risk preference. To specifically test for this post-hoc, across all participants, we correlated the change in fatigue and effort against our economic metrics of risk preference, ambiguity preference, choice strategy, the rEV r^2^, and the pWIN r^2^, in both the gains and losses domains. We applied a full-Bonferroni correction (*p* = 0.005) to account for these 10 additional tests on each of our measures of individual fatigue and individual effort. None of these factors indicated a relationship that survived the necessary multiple comparison correction.

### Individual effort/fatigue does not relate to individual change in economic metrics

We also post-hoc examined whether individual levels self-reported fatigue or effort (RSME) relate to changes in our economic measures (risk preference, ambiguity preference, the rEV r^2^, and the pWIN r^2^). Within each domain (gains or losses), we applied a full Bonferroni corrected threshold to p = 0.0125 for the 4 tests. None of the 8 correlations were significant.

## Discussion

We examined how cognitive fatigue impacts economic decision making, focusing on uncertainty preferences (risk and ambiguity) and choice strategies in both the gains and losses domains. We found no mean shifts in preferences or strategies across either domain. Rather, post-hoc analyses revealed that cognitive fatigue resulted in decreases in test-retest stability across both risk preferences and choice strategies.

### Cognitive fatigue reduces the stability of risk preferences

We found no mean shifts of uncertainty preferences (risk or ambiguity) due to cognitive fatigue. Rather, cognitive fatigue reduced the stability of risk preferences. When fatigued, subjects were inconsistent in their risk preferences, demonstrating more variable risk attitudes.

Multiple studies have suggested that suboptimal mental states are associated with increased intra-individual variability in performance measures such as reaction time [32–34]. Only one study, by Levy and colleagues (2013), has previously reported on altered within-subject variability in risk preferences due to a state manipulation, finding that food deprivation led to decreased variability in risk preferences [35]. It is likely the case that alterations in decision making due to cognitive fatigue and food deprivation occur through different mechanisms, and it is interesting to see opposite effects on choice variability. These results highlight the importance of considering alterations in intra-individual variability in addition to the standard mean shifts.

In contrast to increased instability in risk preferences, we found no changes in ambiguity preferences. This finding appears to concur with neurobiological evidence that these two preferences may be dissociable [36]. However, it is not clear if our results should be taken as further evidence of such dissociation, as we also found weaker test-retest reliability for ambiguity within the control group (greater within-subject variability for ambiguity preferences), which would have limited our power for detecting alterations due to cognitive fatigue (see Table 4).

### Cognitive fatigue reduces the stability of choice strategy

In both the gains and losses domains, fatigue reduced the stability of choice strategy without leading to mean shifts (i.e., a general shift towards more satisficing or maximizing behaviour). It is quite interesting that this result mirrors the alterations we found for risk preferences, as risk preferences and choice strategy are not correlated across our participants, and ostensibly measure different aspects of decision making.

Of note, reduced effort in decision making is potentially an attractive explanation for how cognitive fatigue increased the noise in our choice strategy metric. However, such a motivational change would actually have presented as an increase in satisficing behaviour (decreased choice strategy)–exactly the shift that we initially hypothesized and did not find. Rather, participants continue to, on average, employ the same relative levels of satisficing and maximizing strategies in their choice behaviour, suggesting that they are maintaining their behavioural motivation in our incentive compatible tasks.

### Implications and relation to a neural network of fatigue

Cognitive fatigue results in greater variability in both risk preferences and choice strategies, which will result in decreased consistency in the actual choices made. Insofar as choice quality can be simply judged based upon whether you would repeat your choice given the same options, these results indicate that cognitive fatigue reduces choice quality.

In relation to economic theory, inconsistent preferences or strategies may lead to failures of transitivity (if we prefer A to B and B to C, we should choose A over C) a key rule of rational choice behaviour [37]. The implications of these results are that decisions made under cognitive fatigue are likely to result in more variable choices than those performed under a non-fatigued state, with the potential for regret when the chooser returns to a rested state. Concurring, in the field of strategic management ‘judgment quality’ describes the effectiveness of decisions with reduced response consistency indicating reduced judgment quality, and erratic decisions have been associated with less optimal results and economic inefficiency [38–40]. That individual alterations are unsystematic and unpredictable consequently make it harder to insure against these uncharacteristic and capricious decisions.

Interestingly, our results resemble the behavioural effects of ventromedial prefrontal cortex (vmPFC) damage, which leads to inconsistent or erratic preference judgements without a slowing of response times [41,42]. This suggests that cognitive fatigue has a transient effect that results in alterations of behaviour in ways similar to those with physical damage to their vmPFC. The vmPFC is further implicated in the production or subjective experience of cognitive fatigue, as individuals with focal lesions in the vmPFC reported significantly greater levels of fatigue as compared to individuals with lesions in other locations within the prefrontal cortex (PFC) [43].

### Maintained N-back task performance despite cognitive fatigue

Participants in the fatigue group maintained a high level of performance on the N-back task despite reporting increasing levels of fatigue and indicating that high levels of mental effort were required to perform each subsequent block.

This observed pattern of unchanged task performance under cognitive fatigue is not uncommon [44]. Surprisingly, despite the reliability in observing fatigue carry-over effects, primary time-on-task decrements are far less consistently seen, with success in inducing fatigue usually determined by its effects on the secondary task [11,20,45]. It is unclear whether this discrepancy may be mediated by the demands of the task that is used to induce fatigue. Shigihara and colleagues (2013) reported that while task performance was not diminished in 30 min of a 2-back task, performance decreased over time when participants performed the relatively easier 0-back task for the same amount of time [22]. The authors suggest that mundane tasks may increase sleepiness, while more challenging tasks paradoxically increase motivation and lead to maintenance of high levels of performance. A future challenge may be to clarify which types of cognitive tasks are most affected by fatigue and to construct a unifying explanation of why certain tasks reveal a time-on-task effect while others remain impervious to fatigue.

### Relating Cognitive Fatigue to other state alterations

It is unclear how cognitive fatigue relates to other state alterations, such as sleep deprivation, aging, or ego-depletion. A fruitful area for future investigations would be to investigate how various state alterations may share common mechanisms.

A priori, one may expect that there would be similarities between the effects of cognitive fatigue and sleep deprivation. In fact, there is a recent model of sleep deprivation effects, called ‘the state instability hypothesis’, whose name sounds strikingly similar to our found effects [46]. This model posits that sleep-initiating mechanisms disrupt a person’s capacity to maintain alertness, resulting in ‘lapses’ that occur briefly and are interspersed with otherwise normal performance capabilities. Such a model cannot account for the effects of our study, as the hypothesized effects would have been apparent in two ways in the comparison of the cognitive fatigue group to the control group, both of which were absent, 1) reduced r-squared values for either the rEV or pWIN (or both) and 2) reduced response times.

Our cognitive fatigue manipulation involved 60 to 90 minutes of a strenuous task that engaged executive control. A similar methodology, although involving only ~10% of the manipulation duration, occurs in ego-depletion manipulations [47]. In ego-depletion studies, following the manipulation task, participants are presented with situations or tasks in which they would normally exercise willpower to exert self-control over their behaviour. It is unclear how to conceptually relate cognitive fatigue and ego-depletion, but given the similar methodologies, it is interesting to consider their potential overlap. Baumeister (2002) hypothesized that ego-depletion should result in more impulsive consumer behaviours. No prior studies have tested this experimentally [48]. Potentially, our current study may be considered to test and disprove this hypothesis. We note that there is controversy about the effectiveness of the ego-depletion manipulation, with the potential that participants’ beliefs strongly influence the effect [49,50].

### Future directions

Of specific interest for future studies will be examining how cognitive fatigue may dissociably alter sub-components of the cognitive processes engaged during economic decision making. As we find a general effect across economic decision making metrics, this suggests that cognitive fatigue is altering cognitive processes that are engaged across these economic metrics. Possible examples include interfering with working memory, preventing memory consolidation of prior choices, interfering with the application of preferences, or interfering directly with the comparison process.

There is potentially great utility in examining and comparing how varied state modulations commonly and dissociably alter economic decision making. For example, in these same tasks, we recently showed that aging produces specific shifts of risk preferences and strategies in the losses domain, without altering these processes in the gains domain [16].

The duration of our cognitive fatigue manipulation (60 to 90 minutes) was specifically aimed at examining a duration that is common in everyday life. It is unclear if longer durations of cognitive fatigue will result in specific shifts in economic preferences, such as enhanced/reduced risk aversion or reduced use of maximizing information. However, we saw no significant differences between the 5-blocks and 7-blocks cognitive fatigue groups (60 and 90 minutes, respectively).

## Conclusions

The results of this study show that cognitive fatigue results in destabilization of risk preferences and the informational strategies participants employ. This increased variability in choice behaviour can undermine the integrity of decisions, resulting in diminished choice behaviour quality.

## Supporting Information

S1 Datafile Data file. (XLSX)
