# Correction: Cognitive Fatigue Destabilizes Economic Decision Making Preferences and Strategies

**DOI:** 10.1371/journal.pone.0138589

**Published:** 2015-09-14

**Authors:** The PLOS ONE Staff

The order of Figs 2 and 3 is switched. Please view the correct Fig 2 and Fig 3 here. The publisher apologizes for the error.

**Fig 2:**
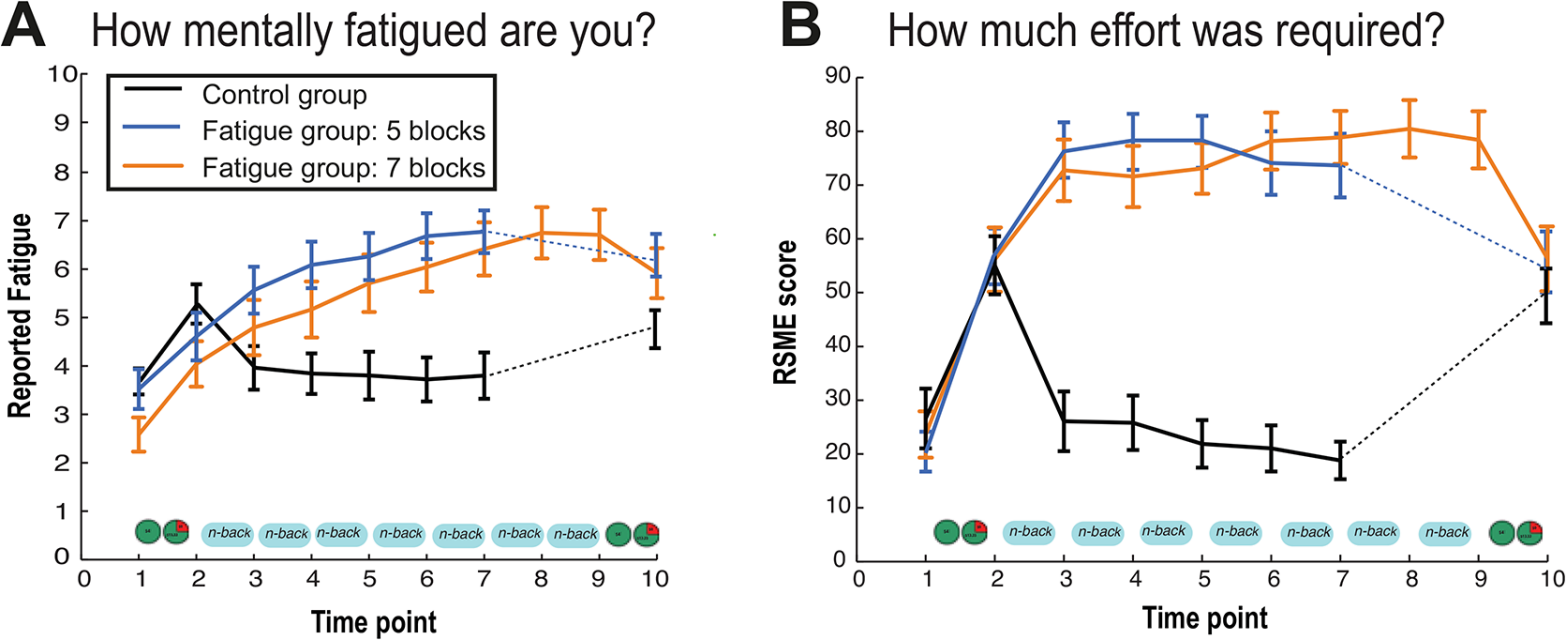
Cognitive fatigue ratings. State fatigue and effort across the experimental protocol. The fatigue (orange bars) and control groups (light and dark blue bars: 5-blocks and 7-blocks of N-back respectively) did not differ significantly in **(A)** self-reported cognitive fatigue pre- manipulation and **(B)** RSME scores, at baseline. However, post- manipulation, the fatigue group reported significantly higher cognitive fatigue and RSME scores as compared to the non-fatigue group, suggesting that the manipulation was successful in inducing fatigue in the fatigue groups.

**Fig 3:**
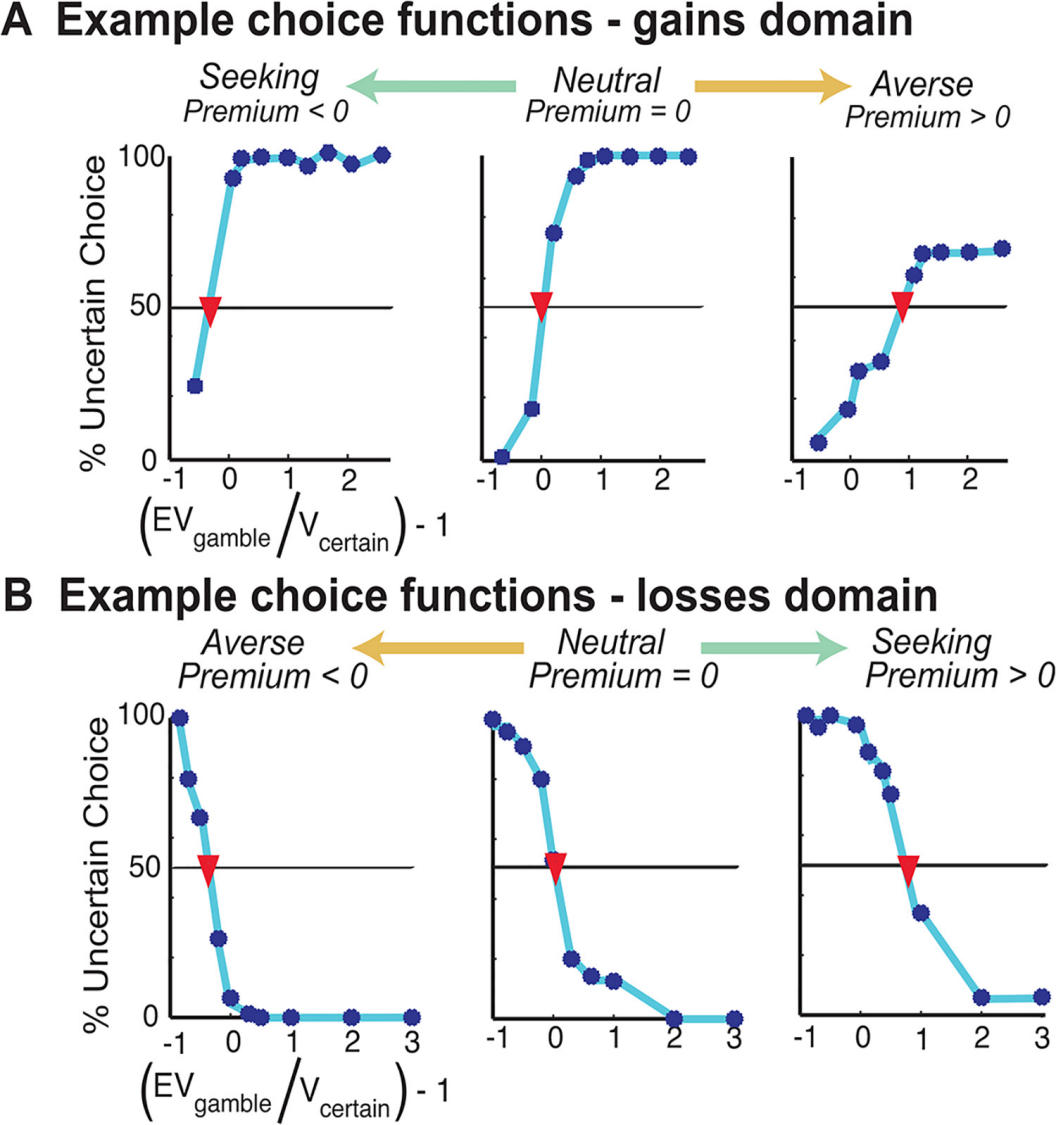
Example choice functions. **(A)** In the gains domain, the range of risk preferences is represented on a continuum from risk seeing (left) to risk averse (right). The indifference point of each choice function is marked with a red inverted-triangle. Risk premium is determined by the value on the ‘(rEVG / Vc) -1’ (x-axis) at this indifferent point. **(B)** In the losses domain, the range of risk preferences is represented on a continuum from risk averse (left) to risk seeking (right).
